# Conflict and exchange rate valuation: Evidence from the Russia-Ukraine conflict

**DOI:** 10.1016/j.heliyon.2023.e16527

**Published:** 2023-05-31

**Authors:** Jianhua Xu, Khalid Khan, Yang Cao

**Affiliations:** a Anyang Institute of Technology Anyang, Henan Province, China; b College of Finance, Qilu University of Technology, Jinan, Shandong, China

**Keywords:** Exchange rate, Ukraine conflict, Causal inference, Conflict, Counterfactual predicting

## Abstract

The Russian-Ukraine conflict is decisive in determining the course of contemporary global politics. The war has a spillover effect, with potentially considerable direct and indirect consequences on economic activity. Therefore, the study evaluates the causal impact of the Russian-Ukraine conflict on the exchange rate of Russia. The results explore that the conflict has a negative effect on the exchange rate and observed rapid depreciation. The outcomes show a rapid divergence from counterfactual predictions, and the actual exchange rate is consistently lower than would have been expected in the absence of conflict. The point-wise causal effect displays an estimate of the exchange rate depreciation following the conflict. In relative terms, the response variable decrease, suggesting that currency depreciation is observed during the intervention period. The counterfactual prediction provides a robust technique for evaluating the size of shocks and can be used as a reference to estimate the genuine impact of the conflict in various ways.

## 1 Introduction

The movement of capital and the balance between exports and imports determines the exchange rate. The occurrence of economic instability and geopolitical uncertainty due to conflicts and civil unrest can significantly impact investors and financial stability [1]. These events are crucial in shaping the course of modern geopolitics [2,3]. Moroever, the trade flows are an essential channel for currency behaviour and have been linked with resources such as oil and gas [4]. The phenomenon is significant for Russia, which possesses abundant natural resources [5]. The country has a distinct role in the global economy and geopolitics, and its instability has serious repercussions. Likewise, trade flows play a vital role in determining the exchange rate of the ruble (RUB) due to Russia's position as a major exporter of oil. The value of the RUB is intricately tied to the price of oil, which sees an increase as the oil price rises [6]. Although the RUB benefited from rising oil prices during the first half of 2020, the conflict caused significant economic turmoil and prompted sanctions that disrupted trade flows. As a result, oil prices soared to above $130 a barrel, and gas prices reached record highs [7], which were reasoned for the stability of the RUB. However, Russia experienced significant economic turmoil due to the conflict, which prompted sanctions and disrupted trade flows [8]. These sanctions substantially affect the country's energy exports, likely impacting the RUB's value.

This study evaluates the causal inference of the Russia-Ukraine conflict on the RUB exchange rate. The study highlights that due to geopolitical uncertainty and the imposition of sanctions, the currency has experienced a loss in value. Currency risk has been a major concern in this regard. However, in the pre-conflict period, the RUB remained stable, and Russia experienced a significant current account surplus in 2020–2021 caused by the trade surplus. Likewise, exports of oil and natural gas bolstered the financing of imports while also upholding the stability of the national currency and financial system. Furthermore, the conflict has economic ramifications [9] and may cause an imbalance in Russia's currency and balance of payment [10]. As a result, Russia has attempted to enhance its currency independence by denominating transactions in non-Western currencies and fostering financial relationships with partner nations to build a de-dollarized financial system. Moreover, several countries have initiated a reassessment of their supply chain strategies to reduce energy supply disruption. Meanwhile, European Union (EU) countries intend to reduce their reliance on Russian gas and oil supplies. Similarly, the conflict is expected to alter geopolitical dynamics, potentially leading to a shift toward regionalization instead of globalization [11]. Consequently, multinational corporations review their operational strategies and positions favoring or against political or economic organizations when disputes destabilize the world market and financial system.

Russia invaded Ukraine on February 24, 2022, which resulted in several political decisions and market changes that generated geopolitical uncertainty [12]. On the eve of the invasion, the RUB was trading at a rate of 81 to the dollar, falling to 150 by early March. In addition, the stock market has been closed since February 25, 2022, and the value of the RUB has hit a historic low. This has led to the perception that the country is “uninvestable for foreign investors.” The sanctions were imposed to ban the import of Russian oil, liquefied natural gas, and coal to the U.S. Furthermore, investment in the energy sector was restricted, targeting the Russian government and businesses. The EU member states devised a plan to reduce their dependence on Russian energy, and the UK pledged to gradually discontinue its import of Russian oil. Furthermore, the G7 removed Russia's designation as the “most favoured nation.” The intention was to isolate Russia from international financial markets and create difficulties in financing the conflict [13]. The sanctions cut off numerous Russian banks from the global interbank payment system and restricted western banks from conducting business with Russian banks [1]. Furthermore, the sanctions blocked the Russian central bank's accounts denominated in allied currencies, leaving half of the central bank's foreign exchange holdings inaccessible. The sanctions sought to cut Russia off from the world economy; the RUB has lost more than half its value. In response, the Russian central bank implemented stringent capital restrictions on February 28, 2022, which maintained the value of the RUB and avoided a currency crisis.

Meanwhile, the interest rate increased from 9.5% to 20% to support the RUB. Consequently, raising interest rates appeared to have stopped capital flight and helped stabilize the RUB. Meanwhile, sanctions resulted in limited access to foreign financing. Moreover, capital flight pressures were reduced since investing in Russian assets was more appealing due to the higher return. The government also implemented capital control laws to prevent the currency from devaluing. In this regard, citizens were prohibited from transferring foreign money overseas and faced a stringent six-month cap on the amount of foreign currency withdrawn from Russian banks. Furthermore, Russian enterprises receiving foreign currency money must convert 80% of those earnings for RUB with the central bank. Russian exporters of products and services must convert 80% of their foreign currency earnings into RUB, boosting their value. Furthermore, to capitalize on the increased energy demand, Russia ordered foreign firms acquiring oil and gas from Russia to have a specific account with a Russian bank, through which payments denominated in foreign currencies will be converted to RUB before paying suppliers. As a result, the RUB recovered from its low point due to these market intervention strategies. The currency and financial system averted collapse following economic and financial restrictions, and the currency recovered its pre-invasion level [10]. Thus, the discussion about the ongoing conflict on the Russian exchange rate is critical in determining and estimating the conflict's perils. This study assumes a situation of pre-and post-intervention observing the difference between the actual and the predicted values. In simple words, the study evaluates the scenario if there is conflict and vice versa and its consequences in relative terms.

The study represents a valuable addition to the current literature. Specifically, the paper compared and established that the conflict between Russia and Ukraine has had a negative impact on the RUB exchange rate. Additionally, the study highlights the harmful effects of this conflict on the RUB exchange rate, which must be comprehensively understood and resolved. To the best of our knowledge, no prior research has investigated the cause behind the conflict's influence on the RUB exchange rate. The conflict has been disruptive news, leading to a significant market reaction and a sharp decline in the exchange rate. The market's response was extreme, resulting in a sustained period of depreciation for the RUB, with the exchange rate remaining at its lowest level. If there had been no conflict, the RUB exchange rate would not have depreciated. Therefore, the findings of this study hold great significance as a benchmark for countries facing similar challenges. Furthermore, the study presents a more reliable method for measuring the impact of conflict on the RUB exchange rate. The empirical design of counterfactual prediction takes into account the role of exogenous variables, which is an important consideration. The research findings suggest that the Russia-Ukraine conflict has negatively affected the RUB exchange rate. As counterfactual projections often quickly diverge from reality, the actual RUB exchange rate regularly fell below what would have been expected in the absence of conflict. Based on the point-wise causal effect, the RUB exchange rate has reached its highest level. The study demonstrates that evaluating the magnitude of shocks during emergencies through counterfactual prediction-based causal effect assessment can be a concise and reliable technique. This approach may serve as a reference point for estimating the actual impact of the Ukraine conflict in various aspects.

The study comprises the literature review in section 2, followed by the causal inferences approach in section 3. The data is elucidated in section 4. Section 5 describes the results and concludes in the last section.

## 2 Literature review

The literature has studied the response of the exchange rate to different conflicts and social uprisings. Most of the studies concluded that conflict negatively impacted exchange rate. For example, Warburton [14] showed that the U.S. dollar depreciated in the short term around the Iraq War. Odhuno [15] examined the civil conflict's impact on exchange rate fluctuations. The finding suggested that rebel attacks resulted in currency depreciation while government operations caused appreciation. Dreger et al. [16] exmained the effcet of Russia-Ukraine conflict in 2014 on the RUB exchange rate. In addition, the findings revealed that conflict has resulted in sanctions which amplified the depreciation of the RUB. Moreover, the oil prices and sanctions were related to RUB volatility. Duarte et al. [17] showed that conflict considerably affected exchange rates during the first world war. Cheung [18] examined the Syrian conflict's impact on the macroeconomic indicators and concluded that civil war negatively impacts the well-being of citizens. Lemaire [19] showed that civil disputes led to exchange rate overvaluations for Arab league member countries. Michail [20] evaluated the different civil conflict effects on the exchange rate in developing economies. The outcomes explored the evidence of depreciation caused by macroeconomic factors. On the contrary, international conflicts have no impact on the exchange rate.

The existing literature consists of studies investigating the Russia-Ukraine conflict's effects on the exchange rates of different countries. The outcome of most of the studies confirmed that ongoing conflict had depreciated the currencies. In this regard, Chortane and Pandey [1] examined the response of global currencies to the Russia-Ukraine conflict. The findings revealed the negative impact of this conflict on currencies. It further confirmed that the Russian rouble depreciated while pacific countries' currencies appreciated. However, the Middle East & African currencies were insignificant. Aliu et al. [21] showed that the EUR/RUB significantly influenced the Euro devaluation. Moreover, the exchange rates have a long-term association during the conflict. Lyócsa and Plíhal [22] investigated the Russo-Ukraine crisis on the USD/RUB price fluctuations and the EUR/RUB exchange rates. The outcome explored that the crisis impacted the price fluctuations of the two exchange rates. Sohag et al. [23] demonstrated that the RUB appreciated in response to the rising oil prices and trade, while depreciation was caused by economic uncertainty. Guo and Chen [24] found that tariff restrictions profoundly impacted the Renminbi (RMB) exchange rate fluctuations. Similalry, the positive and negative events significantly caused the appreciation and depreciation of the RMB, respectively. De Groot and Skok [25] found that the Russian invasion resulted in mass migration and pressure on the Ukraine currency. Caldara et al. [26] explored that uncertainty caused by the conflict led to currency depreciation. Moreover, geopolitical uncertainty had adverse risk sentiments and may trigger international capital flows. Będowska-Sójka et al. [27] investigated the influence of the Russian-Ukraine crisis on several asset classes. The findings showed asset classes have uneven risk sensitivity in time and frequency domains. Aziz et al. [28] scrutinized the impact of oil prices on exchange rates and discovered that demand shocks cause rates to rise in net oil-producing nations. Bossman et al. [29] confirmed that the Russia-Ukraine conflict significantly influences major currencies, particularly at low and high extremes. Feng et al. [30] examined the influence of geopolitical risk on capital flows and explored that capital flows in advanced and emerging nations face considerable reductions when geopolitical risk rises.

The current studies about the conflict impact on the exchange rate comprised of the causality impact on the exchange rate fluctuations. However, given the macroeconomic context, estimating the causal effect of conflict on the exchange rate may be more difficult. This is because the causal inference is a complex process rather than a simple link. The influence of the Russia-Ukraine war conflict on exchange rates was examined in several recent research. The majority of these research, however, favoured correlation rather than causal inference. Some studies have used event studies to evaluate the effects of conflicts on exchange rates. The event research technique is frequently employed in earlier literature, but it has the issue of measuring the impact of disasters before and after the event on the exchange rates. However, comparing the two times does not equal the causal effect. Furthermore, studies used time series data to examine how conflicts affected exchange rates. However, the abovementioned methodologies cannot directly estimate the causal influence since causal assessment frequently necessitates a thoroughly thought-out counterfactual context [31]. The preceding literature includes research on the influence of conflict on various exchange rate behaviour. However, most of these studies have focused on a single country's war or conflict and its consequences on other countries worldwide. It suggests that the conflict may have a diverse impact because the countries may not be bordering, and other variables may influence exchange rate movement. There is a scarcity of studies on the war conflict and its effect on the source country of the conflict in contemporary literature.

Furthermore, studies have been conducted assuming conflict has broken out, and the exchange rate reaction reflects these uncertainties. However, if no conflict occurs, the preceding literature fails to assess the issue. This allows you to compare the observed and anticipated values and provides detailed information about the conflict effect. Moreover, most previous research used conventional techniques to identify the impact of conflict on the exchange rate. However, no studies have employed methodology to identify causal inferences in the literature. The current study thus fills the gap described above in the earlier investigations. Furthermore, the influence of the Russia-Ukraine conflict on the RUB is considered, which may provide comprehensive information on the conflict's direct impact on the RUB exchange rate in Russia. This analysis also considers how the RUB would react without conflict. This includes comparing the actual results with the expected and detailed information regarding the conflict's impact. Likewise, the present research used the causal inference technique to generate exact counterfactual predictions based on the untreated control time series. As a result, the study offers important information to the relevant parties.

## 3 Data and methodology

The methods, such as the difference-in-difference (DD), are used to analyze causal effects. However, the method has certain limitations when interpreting the causal effect. These shortcomings are summarized in the following manner. Firstly, the process assumes that the data is i.i.d., as DD is based on a static regression model, even though the design has a temporal component. Hence, when fitted to serially correlated data, it tends to produce positive conclusions with narrow uncertainty ranges [32,33,34,35]. Secondly, the technique only considers the effect before and after the intervention, neglecting the growing influence over time, which is essential in causal inference. Finally, past research has constraints when building a synthetic control from a collection of predictor variables when using time series for DD analysis. In investigating the causal effect, our study avoids such a circumstance [36].

The disadvantages associated with DD methods can be addressed using state-space models to depict the time-based changes of an observable result. Additionally, utilizing a spike-and-slab before the fully Bayesian treatment helps avoid overfitting by integrating the posterior uncertainty related to selecting variables and the degree to which they influence the study's forecasts. As a result, the causal inference approach introduced by Brodersen et al. [37] is employed in the study.

The methodology commences with Bayesian structural time-series models, which are characterized by a set of equations. The foundation of the technique is formed by a set of equations that define Bayesian structural time-series models.

(1) $$x_{t}=Z_{t}^{T}\beta_{t}+\varepsilon_{t}$$

(2) $$\beta_{t+1}=T_{t}\beta_{t}+R_{t}\eta_{t}$$

where $\varepsilon_{t}∼N\left(0,\sigma_{t}^{2}\right)$ and $\eta_{t}∼N\left(0,Q_{t}\right)$ are independent of all other unknowns. The link between observed data $x_{t}$ to a latent d-dimensional state vector $\beta_{t}$ is established in Equation (1). The evolution of state vector at $\beta_{t}$ through time is stated by Equation (2). In this study, $x_{t}$ is a scalar observation, $Z_{t}$ is a *d*-dimensional output vector, $T_{t}$ is a d×d transition matrix, $R_{t}$ is a d×q control matrix, $\varepsilon_{t}$ is a scalar observation error with noise variance $\sigma_{t}^{2}$, and $\eta_{t}$ is a *q-*dimensional system error with a q×q state-diffusion matrix Q, where q≤d.

The regression component responsible for producing counterfactual predictions is essential for the examined applications. This is achieved by creating a synthetic control through a combination of markets without treatment. The utilization of observed responses from such marketplaces enables the explanation of variable components in treated markets that more general seasonal sub-models cannot readily capture.

The method used in this study to generate accurate predictions about hypothetical scenarios relies significantly on using control time series that have not been subject to any interventions. The reason for this is that these time series account for shared variance components among the series, including the effects of other factors that the model would not typically take into consideration. One way to incorporate these control time series into the model is through linear regression. The coefficients in the model can either remain constant or vary over time. In this research, we introduce contemporaneous variables with stable coefficients, which can be formulated in state-space notation form by setting $Z_{t}=\alpha^{t}x_{t}$ and $\beta_{t}=1$.

Let designate the group of all model parameters as ϑ and use $\beta =\left(\beta_{1,\ldots \ldots \ldots ..,}\beta_{m}\right)$ to denote the complete state sequence. Brodersen et al. [37] propose modifying a Bayesian method of inference by supplying a prior distribution ρ(θ) for the model parameters and a distribution $\rho \left(\beta_{0}\vartheta \right)$ for the initial state values, and sampling from $\rho \left(\beta ,\vartheta_{x}\right)$ through the Markov Chain Monte Carlo.

Concerning estimating the impact in a pointwise manner in Equation (3) as below;

(3) $$\vartheta_{t}^{\tau }≔x_{t}-∼x_{t}^{\tau }$$

A construction is made for each draw τ and for each time point =n+1,……,m, where n represents the time of treatment, to gather findings related to the a posteriori causal effect. It is also possible to estimate the cumulative impact of the intervention over time.

This calculation involves adding the causal increments to arrive at the cumulative sum in Equation (4) as follows;

(4) $$\sum_{t=n+1}^{t}\vartheta_{t}^{\tau }\forall t=n+1,\ldots \ldots .,m$$

**Fig. 1:**
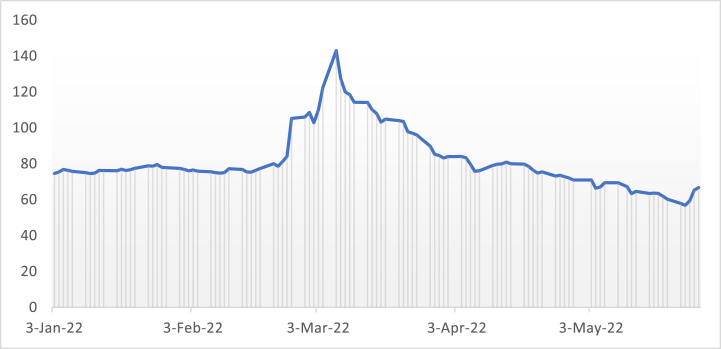
The trend of the RUB exchange rate during the conflict.

This study uses the daily exchange rate from 2022:01:01 to 2022:05:31. The sample period is critical regarding the geopolitical instability caused by the conflict, reflected in the international economic cost and particularly to Russia. The RUB exchange rate is the daily spot rate against the dollar, derived from the Federal Reserve Economic Data (FRED). As a result, the article selects the outbreak of conflict on February 24, 2022 as the starting point. Fear and uncertainty have gripped the world's financial markets as a result of the conflict. Therefore, we set the pretreatment period from 3rd January to February 23, 2022 and the posttreatment period from 24 February to May 31, 2022. Fig. 1 depicts the RUB trend during the conflict. It implies that the RUB depreciates against the dollar. The rate declined from 78.598 on February 24 to 143 on March 7, accounting for more than 60% depreciation. However, it regains its pre-conflict position in the last week of April.

**Fig. 2:**
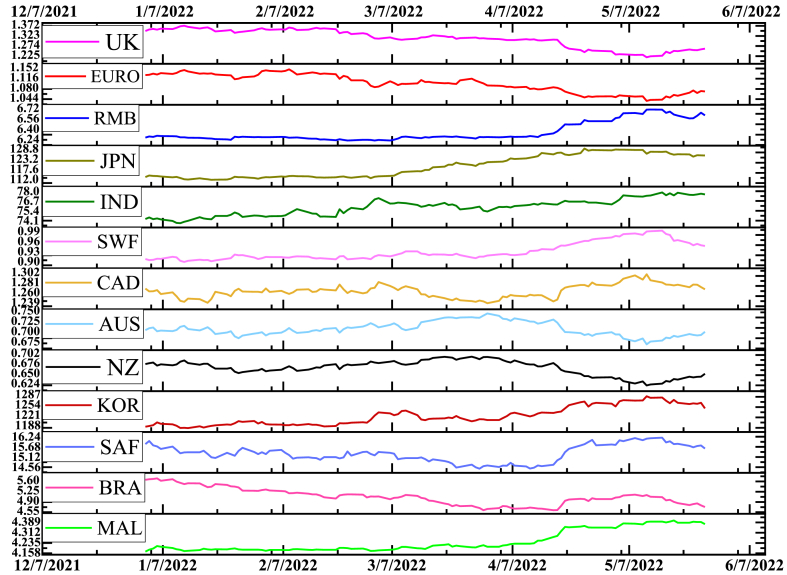
The trend of the RUB exchange rate during the conflict.

The study undertakes 13 exchange rates worldwide as a covariate to investigate the causal inference of conflict. These series correlate with our outcome of interest while not directly affecting the treatment, making them excellent predictors (Brodersen et al., 2015). The covariate includes the Australian dollar (AUD), Brazil Real (BRL), Canadian dollar (CAD), Chinese Renminbi (CNY), Euro Zone (EUR), Indian Rupee (INR), Japanese Yen (JPY), Korean Won (KRW), New Zealand dollar (NZD), Malaysian Ringgit (MYR), Switzerland Franc (CHF), UK pound (GBP) and South Africa (ZAR). The data of the covariates are derived from the FRED. Fig. 2 illustrates the behaviour of these covariates over the period. It exhibits that these currencies are unaffected by the conflict and continue their normal behaviour. Meanwhile, the RUB exchange rate has shown an upward trend against the dollar or depreciated enormously. On the contrary, the major currencies act as control variables with no abnormal movements, which suggests that the Russia-Ukraine conflicts have no direct impact.

The summary statistic is highlighted in Table 1. It shows a larger difference between maximum and minimum values for RUB, suggesting greater volatility. The standard deviation supports the higher volatility. On the other hand, the skewness values are positive for most of the currencies. Similarly, the kurtosis values reveal platykurtic distribution for all currencies rate except RUB, RMB, and CHF. Finally, the Jarque Bera values confirm that most variables are non-normally distributed.

**Table 1:** Summary statistics.

	Mean	Maximum	Minimum	Std. Dev.	Skewness	Kurtosis	Jarque-Bera
RUB	81.965	143.000	56.800	16.355	1.353	4.623	42.753***
AUS	0.723	0.760	0.685	0.016	0.233	2.338	2.814
BRA	5.079	5.703	4.617	0.289	0.370	2.368	4.058
CAD	1.270	1.306	1.245	0.013	0.197	2.638	1.226
CHF	0.940	1.003	0.910	0.025	1.130	3.056	21.932***
EURO	1.100	1.149	1.038	0.031	−0.268	1.872	6.697***
IND	75.826	77.820	73.770	1.071	0.006	2.057	3.814
JPN	120.827	130.940	113.720	6.020	0.338	1.455	12.210***
KOR	1224.559	1288.650	1186.960	8.890	0.637	2.112	10.352***
MAL	4.244	4.404	4.171	0.079	1.023	2.307	20.030***
NZD	0.669	0.698	0.624	0.019	−0.614	2.501	7.547***
RMB	6.433	6.788	6.308	0.145	1.296	3.066	28.844***
ZAR	15.295	16.190	14.478	0.461	0.059	2.108	3.473
UK	1.312	1.372	1.220	0.043	−0.573	2.130	8.880***

Note: *** denotes a significance level at 1% level.

## 4 Empirical result

**Fig. 3:**
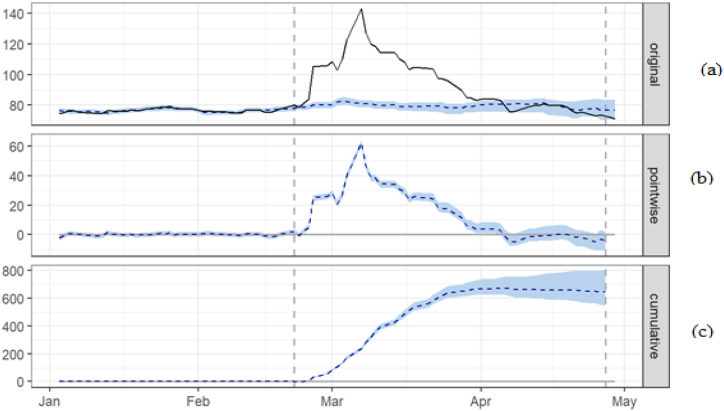
The time-varying causal effect of conflict on the RUB. Note: (a) the dotted line is the counterfactual forecast value, (b) pointwise (daily) incremental impact of the RUB, and (c) cumulative impact of the RUB.

The results of the causal inference taking into account control variables are shown in Fig. 3. The solid line shows data, while the dotted line shows the counterfactual forecast for the time after treatment. Similarly, the pointwise causal effect is shown in the second panel. Finally, the intervention's overall impact is plotted in the third panel. Moreover, the treatment dividing line is represented by the vertical dashed line. The model achieved a good match for RUB prior to the treatment dates. In the absence of treatment, it consists of original data and counterfactual estimates. Thus, the model is estimated in the pre-period to establish a link and employed in the post-period, with the prediction providing a counterfactual assessment. It deviates sharply from counterfactual forecasts, with the actual RUB consistently upper than what would have been predicted in the absence of conflict. As a result of the RUB's record low value, the country is uninvestable for international investors. The sanctions were implemented to prohibit imports, isolate Russia from global financial markets, and make it more difficult to finance the conflict [13].

The two curves indicate a resumption of a trend in the RUB exchange rate in April. This suggests that the RUB responded significantly to the uncertainty caused by the conflict and reached a low level. The causal effect at individual points explains the difference between the observed and expected data. It remains around zero before increasing rapidly and then decreasing after the action is taken. Panel two presents an estimation of the devaluation of the RUB after the conflict and shows that it reached its peak in mid-March. To stabilize the RUB, Russia's central bank increased interest rates and imposed capital restrictions. The government also implemented capital control laws to prevent currency devaluation. As a result, citizens are not allowed to transfer foreign money overseas and are subject to a strict limit on foreign currency withdrawals. As a result, Russia's currency and financial system avoided the collapse, and the RUB regained its pre-invasion level. In addition, the Russian central bank's strict control over the currency resulted in an appreciation of the RUB comparable to its pre-invasion level. Panel three shows the causal impact over time to determine the cumulative effect, revealing that the RUB has decreased by 17% in relative terms, indicating a negative impact during the post-treatment period.

The term “Average” refers to the mean value for the period following the intervention, while “Cumulative” indicates the total of individual data points. As per Table 2, the response variable has an average value of approximately 93.41 post-intervention. In contrast, if no intervention had occurred, we would have expected an average response of 79.69. This hypothetical prediction has a 95% confidence interval of [76.39, 81.81]. By subtracting this hypothetical value from the actual observed response, we can determine the causal effect of the intervention on the response variable. This effect has a 95% confidence interval of [11.59, 17.02]. The response variable has an overall value of 4.39K by adding all the individual data points for the post-intervention period. On the other hand, if there had been no intervention, the total response value would have been 3.75K, and this prediction's 95% confidence interval ranges of [3.59K, 3.85K].

**Table 2:** The average causal effect of the Ukraine war conflict on the RUB.

	Average	Cumulative
Actual	93	4390
Prediction (s.d.)	80 (1.3)	3745 (60.4)
95% CI	[76, 82]	[3590, 3845]
Absolute effect (s.d.)	14 (1.3)	645 (60.4)
95% CI	[12,17]	[545, 800]
Relative effect (s.d.)	17% (1.6%)	17% (1.6%)
95% CI	[15%, 21%]	[15%, 21%]

Note: The ‘Average’ column discusses the average (across time) post-intervention period. The ‘Cumulative’ column sums up individual time points. The number of MCMC samples to draw is 5000.

The above results are stated in absolute numbers. The response variable showed an increase of 17% in relative terms, with a 95% confidence interval of +15% to +21%. Therefore, the positive impact observed during the intervention period is statistically significant and is unlikely due to chance occurrences. However, it is essential to note that to determine if this increase is also substantial, one must compare the absolute effect (13.72) with the intended outcome of the intervention. The likelihood of achieving this effect is very low, indicating a significant causal impact. The findings suggest that the RUB exchange would not have depreciated if the conflict had not occurred.

## 5 Conclusion

The study inspects the causal effect of the Russia-Ukraine conflict on the RUB exchange rate. Moreover, the study investigated the difference between the observed and the predicted values due to intervention. The study evaluates the condition if there is conflict and vice versa and its consequences in relative terms. The control variables in major exchange rates were used as covariates, which correlate with the RUB exchange rate result but are not affected directly by the treatment. The outcome explored that the Ukraine conflict had a negative impact on the RUB exchange rate and observed rapid depreciation. The results show rapid divergence from counterfactual predictions, and the actual RUB exchange rate was consistently upper than would have been expected in the absence of conflict. The point-wise causal effect displays an estimate of the Rub exchange rate depreciation following the conflict and reaching the bottom. The cumulative causal effect adds up to the causal effect over time. In relative terms, the response variable increased, suggesting that currency depreciation is observed during the intervention period. The results established that the RUB exchange would not have lost value if the conflict had not broken out. The studies of Warburton [14]; Odhuno [15]; Duarte et al. [17]; Cheung [18]; Lemaire [19], and Michail [20] support the results of this study, which explained that different civil conflicts and conflict has a negative impact on the exchange rate and explored the evidence of depreciation. Similary, in the case of Russia, some studies, such as Dreger et al. [16], examined the Russia-Ukraine conflict in 2014 on the RUB exchange rate and revealed that the war resulted in sanctions which amplified the depreciation of the RUB. This argument is further confirmed by the studies of Chortane and Pandey [1], Aliu et al. [21], Lyócsa and Plíhal [22] and Sohag et al. [23] examined the response of global currencies to the Russia-Ukraine conflict and findings reveal the negative impact of conflict on currencies. Furthermore, the uncertainty caused by the conflict leads to currency depreciation. Moreover, geopolitical uncertainty has adverse risk sentiments and may trigger international capital flows.

The paper makes various policy recommendations. First, multiple sectors of the economy, such as governments and investors, should have access to market information and monitor the impact of conflict on the exchange rate. Investment choices and policy revisions should be based on the conflict's evolution and changes. Additionally, the study promotes the creation of optimal portfolios and the advancement of portfolio diversification strategies throughout the conflict and post-conflict periods. Second, the sanctions have reduced the available investment alternatives, which limits the potential for diversification. The restrictions also reduced overseas investors' investment possibilities, which harmed the Russian exchange rate. As a result, lifting sanctions is expected to result in considerable portfolio investment inflows into Russia. Similarly, the conflict's ending and its contagion effect can successfully mitigate the adverse impact on the exchange rate. Finally, the study shows that causal effect assessment based on counterfactual prediction can provide a succinct and robust technique for evaluating the size of shocks in an emergency. It might be one of the references used to estimate the genuine impact of the Ukraine conflict in various ways. Several future directions can be derived from the current study. For example, the conflict's impact on the regional and most major global currencies can be examined. This might explain how these major and regional currencies fear and uncertainty caused by the Russia-Ukraine conflict. Moreover, currency fluctuations correlate more with trade flows, and trade surplus/deficit can affect currency stability differently. Therefore, the relationship between exchange rates and trade will be interesting. Similarly, Russia is one of the leading energy exporters of oil and gas, and imposing sanctions might disrupt the energy supply and prices. Thus, energy prices can have severe repercussions for currency stability and investigating the relevant relationship can be a valuable contribution to the literature.

## Author contribution statement

.**Jianhua Xu**: Conceptualization, Methodology, Software, Writing original draft. **Khalid Khan**: Visualization, Data curation, Writing original draft preparation. **Yang CAO**: Writing, review and editing.

## Data availability statement

Data will be made available on request.

## Additional information

No additional information is available for this paper.

## Funding

There is no funding source to support this study.

## Declaration of competing interest

The authors declare that they have no known competing financial interests or personal relationships that could have appeared to influence the work reported in this paper.

## Abbreviations

RUB: Ruble
EU: European Union
DID: Difference-in-difference
RMB: Renminbi
MCMC: Markov Chain Monte Carlo
FRED: Federal Reserve Economic Data
AUD: Australian dollar
BRL: Brazil Real
CAD: Canadian dollar
EUR: Euro Zone
INR: Indian Rupee
JPY: Japanese Yen
KRW: Korean Won
NZD: New Zealand dollar
MYR: Malaysian Ringgit
CHF: Switzerland Franc
UK: pound (GBP)
ZAR: South Africa
